# Host-Adaptation of *Francisella tularensis* Alters the Bacterium's Surface-Carbohydrates to Hinder Effectors of Innate and Adaptive Immunity

**DOI:** 10.1371/journal.pone.0022335

**Published:** 2011-07-22

**Authors:** Tiffany M. Zarrella, Anju Singh, Constantine Bitsaktsis, Tabassum Rahman, Bikash Sahay, Paul J. Feustel, Edmund J. Gosselin, Timothy J. Sellati, Karsten R. O. Hazlett

**Affiliations:** 1 Center for Immunology and Microbial Disease, Albany Medical College, Albany, New York, United States of America; 2 Center for Neuropharmacology and Neuroscience, Albany Medical College, Albany, New York, United States of America; Tulane University, United States of America

## Abstract

**Background:**

The gram-negative bacterium *Francisella tularensis* survives in arthropods, fresh water amoeba, and mammals with both intracellular and extracellular phases and could reasonably be expected to express distinct phenotypes in these environments. The presence of a capsule on this bacterium has been controversial with some groups finding such a structure while other groups report that no capsule could be identified. Previously we reported *in vitro* culture conditions for this bacterium which, in contrast to typical methods, yielded a bacterial phenotype that mimics that of the bacterium's mammalian, extracellular phase.

**Methods/Findings:**

SDS-PAGE and carbohydrate analysis of differentially-cultivated *F. tularensis* LVS revealed that bacteria displaying the host-adapted phenotype produce both longer polymers of LPS O-antigen (OAg) and additional HMW carbohydrates/glycoproteins that are reduced/absent in non-host-adapted bacteria. Analysis of wildtype and OAg-mutant bacteria indicated that the induced changes in surface carbohydrates involved both OAg and non-OAg species. To assess the impact of these HMW carbohydrates on the access of outer membrane constituents to antibody we used differentially-cultivated bacteria *in vitro* to immunoprecipitate antibodies directed against outer membrane moieties. We observed that the surface-carbohydrates induced during host–adaptation shield many outer membrane antigens from binding by antibody. Similar assays with normal mouse serum indicate that the induced HMW carbohydrates also impede complement deposition. Using an *in vitro* macrophage infection assay, we find that the bacterial HMW carbohydrate impedes TLR2-dependent, pro-inflammatory cytokine production by macrophages. Lastly we show that upon host-adaptation, the human-virulent strain, *F. tularensis* SchuS4 also induces capsule production with the effect of reducing macrophage-activation and accelerating tularemia pathogenesis in mice.

**Conclusion:**

F. tularensis undergoes host-adaptation which includes production of multiple capsular materials. These capsules impede recognition of bacterial outer membrane constituents by antibody, complement, and Toll-Like Receptor 2. These changes in the host-pathogen interface have profound implications for pathogenesis and vaccine development.

## Introduction

*Francisella tularensis* is an extremely infectious gram-negative bacterium which is readily aerosolized. Inhalation of this Category A select agent can lead to pulmonary tularemia which has a mortality rate of ∼35% in the absence of treatment. Reportedly, antibiotic-resistant strains of this bacterium were developed by at least one nation's biological weapons program [1]. The specter of such an agent being maliciously employed, in concert with the current lack of a licensed tularemia vaccine, has evoked a ground swell of interest in *F. tularensis*.

In addition to being a potentially fearsome biological weapon, *F. tularensis* also is a naturally-occurring zoonotic bacterium found in strikingly diverse environments including an array of warm- and cold-blooded hosts (mammals, insects, arthropods, fresh water protozoans) [1], [2]. The *tularensis* subspecies (ssp., comprised of type A strains and found in North America) is maintained primarily in a terrestrial cycle involving lagomorphs and rodents with transmission via ticks and tabanid (deer, horse, etc) flies. In contrast, the *holartica* ssp. (type B strains which are distributed throughout the northern hemisphere) has a stronger association with water-borne disease and, perhaps consequently, with transmission by mosquitoes (as well as ticks and biting flies). In addition to the broad range of hosts, it has recently become appreciated that within both mammalian and protozoan infection models, the bacterium has both intracellular (replicative) and extracellular (transmissive) phases [3]–[7] suggesting additional environments to which the bacterium must adapt. Furthermore, a growing body of evidence indicates that as *F. tularensis* progresses through the intracellular cycle (phagocytosis and phagosomal escape, cytoplasmic replication, induction of autophagic vacuoles and cellular escape) the bacterium adapts to distinct micro-environments by modulating expression of many genes [8]–[10].

The mutability of this bacterium, which endows such a broad host range, also poses a challenge to vaccine development as the nature of the inoculum (with potentially distinct antigenic compositions) can vary widely. Upon entering the mammalian environment the bacterium will presumably adapt regardless of its immediate history. However, it must be considered that the time required for complete bacterial adaptation of a *F. tularensis* inoculum is currently unknown - as is the impact that this adaption might have on tularemia vaccination strategies. A salient example of how bacterial adaptation can affect vaccination against vector-borne pathogens is provided by the now defunct LYMErix vaccine that induced antibody (Ab) against OspA of *Borrelia burgdorferi*, the tick-borne Lyme disease spirochete. This vaccine was developed largely based on work with non-host-adapted bacteria in which OspA was characterized as an abundant, surface-exposed outer membrane protein which could serve as a protective antigen in syringe-inoculation models of murine borreliosis. After many years of development and commitment of financial resources, it was subsequently revealed that, to be effective, the host's bacteriocidal α–OspA Ab had to enter the feeding tick early in the blood meal to kill the tick-adapted spirochete. This was necessary because, in preparation for exiting the tick and entering the mammalian host, the bacterium dramatically down-regulates expression of OspA. Consequently, any host-adapted (OspA negative) spirochetes that entered the host were “immune” to the host's vaccine-induced immunity [11], an observation which correlated well with the vaccine's sub-optimal efficacy. Production of LYMErix was halted in 2002. The desire to work with host-adapted *F. tularensis* as a platform for vaccine-discovery prompted our previous efforts to recapitulate the bacterium's mammal-adapted phenotype during *in vitro* growth [12].

As *F. tularensis* replicates within host cells, cell-mediated immunity has long been considered paramount to the control of disease. However, the growing recognition that the bacterium has a significant extracellular phase [4], [5], [7] provides a mechanistic explanation for the early observation that adoptively-transferred immune serum provides a degree of protection [13]. This original report has been confirmed and extended under more defined experimental conditions/infection models using both the attenuated type B, live vaccine strain (LVS) [14]-[19] and the human-virulent, Type A strain SchuS4 [20], [21] as challenge inoculums. It should be noted, however, that none of these more recent challenges were initiated with host-adapted bacteria. Where therapeutic administration of either immune serum or Ag-specific Ab has been examined, the serum/Ab's full therapeutic potential was realized by administration beginning no-later than 24 hr post-infection (PI) [16], [20], [22]; further delays in Ab-therapy (potentially allowing for further bacterial adaptation) result in dramatic reductions and/or loss of transferrable protection [16]. Consistent with this susceptibility of non-host-adapted bacteria to Ab, it has recently been shown that animals receiving multiple transfers of immune serum (on days −1, +3, +6, +9, and +12 relative to challenge on day zero) are no more protected from challenge than animals that receive a single equal dose of immune serum one day before infection [21].

In addition prophylactic and/or therapeutic approaches involving immunization with attenuated/inactivated bacteria and/or adaptive transfer of serum/Ab, several purified or semi-purified antigens have been assessed as sub-unit vaccine candidates. Perhaps the most well-characterized is lipopolysaccahride (LPS) which in gram-negative bacterium is readily antibody-accessible – provided the bacterium is not heavily encapsulated. Preparations of *F. tularensis* LPS (a poor TLR4 agonist) have been shown to be protective immunogens by multiple groups [23]–[28]. However, as we and others [25], [29] have observed, the most widely-cited method for preparing LPS from this bacterium (hot-phenol extraction) also co-purifies a significant amount of HMW carbohydrate/putative capsule; this material can amount to 40% of the total “LPS” preparation [25]. This raises the specter that some of the protection ascribed to LPS immunization may, in part, represent anti-capsule responses. Indeed a recent report showed that active immunization with OAg capsule or passive immunization with a mAb directed against the capsular OAg was protective [30].

Our long term interests include the identification of bacterial surface proteins which are both **i)** accessible to host antibodies and/or cellular receptors and, importantly, **ii)** are expressed during mammalian infection. In support of this goal we previously identified *in vitro* culture conditions that recapitulate the bacterium's mammalian host-adapted, extracellular phenotype. Here we describe the further pursuit of our objective which has led us to the discovery that host-adaptation includes multiple changes to the bacterium's surface carbohydrates (LPS and capsules) which cumulatively hinder recognition of OM constituents by Ab, complement, and TLR2.

## Results

### *Francisella tularensis* grown in brain-heart infusion broth display the bacterium's extra cellular, host-adapted phenotype

Host-adapted *F. tularensis* (those replicating in macrophages or in the tissues of mice) display a phenotype distinct from that of bacteria cultivated *in vitro* using standard media such as in Mueller Hinton broth (MHB) or Chamberlain's defined media (CDM) [31], [32]. The phenotypic differences include expression of a distinct bacterial proteome as well as the elicitation of a different reaction from naïve macrophages (MΦ) responding to the bacteria [33]. Recently we reported that growth of *F. tularensis* in BHI induces a phenotype that is indistinguishable from that of bacteria which have emerged from infected MΦ (i.e. extracellular, host-adapted *F. tularensis*) [12]. This work was undertaken in support of our longer-term goal: the direct biochemical identification of surface-exposed, outer membrane (OM) proteins (OMPs) which are expressed during mammalian infection. As candidates for a sub-unit vaccine, such proteins could serve as targets for antibody (Ab) during the bacterium's extra-cellular phase [5], [7].

**Figure 1:**
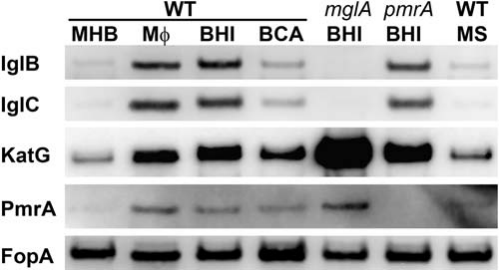
*F. tularensis* grown in MΦ or in BHI are nearly indistinguishable. *F. tularensis* LVS strains were harvested from the culture supernatants of infected macrophages (MΦ) or grown *in vitro* as indicated: MHB (M), BHI (B), MHB supplemented with spermine to 200 µM (MS), BHI supplemented with casamino acids to 17.5 g/L (BCA). Ten micrograms of protein from these bacteria were resolved by SDS-PAGE and transferred to membranes for western blot analysis with antibodies specific to the indicated proteins.

Here, prior to commencing our quest for such OMPs, we first sought to confirm and extend the notion that growth of *F. tularensis* in BHI induces the bacterium's host-adapted phenotype. To this end we used western blot analysis of several differentially-expressed proteins to gauge the similarity between MHB-, BHI-, and extracellular, MΦ-grown *F. tularensis*. To extend our understanding of the regulatory genes and environmental cues which govern host-adaption we also probed BHI-grown, *mglA* and *pmrA* strains along with wildtype (WT) *F. tularensis* grown in the presence of high concentrations of free amino acids [12] (in the form of casamino acids - CA) or spermine [34]. As shown in Fig 1, *F. tularensis* that have emerged from infected MΦ and those which were grown in BHI contain similarly elevated levels of the Intracellular Growth Locus (Igl) proteins B and C, catalase (KatG), and PmrA - a response-regulator protein [35], [36]. These proteins were minimally-expressed by *F. tularensis* grown in MHB (which contains 17.5 g/l casamino acids) or in BHI supplemented with casamino acids to 17.5 g/l (BCA). These protein changes during host-adaptation were entirely dependent on the transcriptional regulator MglA (a homologue of the *E. coli* stringent starvation protein SspA [37] which responds to amino-acid deprivation [38]). MglA activates transcription of the Igls and represses that of KatG [39]. PmrA had a detectable, but less pronounced, contribution to these regulatory changes. Supplementation of MHB with spermine (MS), which induces multiple transcriptional changes in *F. tularensis* [34], had minimal impact on the abundance of these proteins.

### The quest for outer membrane proteins in host-adapted *F. tularensis* reveals putative capsular material(s)

**Figure 2:**
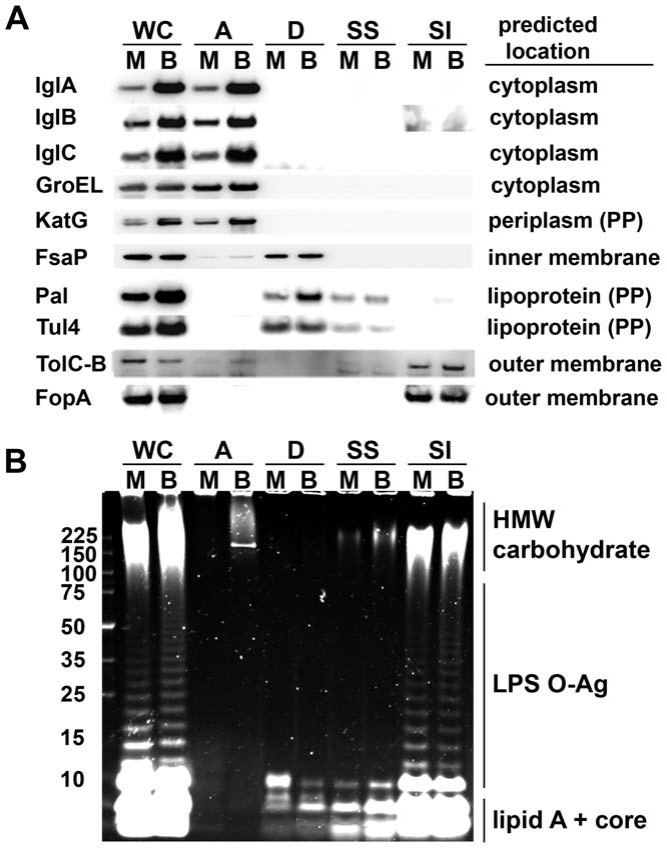
Fractionation of *F. tularensis* reveals the presence of an inducible HMW carbohydrate in host-adapted bacteria. Whole cells (WC) of MHB- (M) and BHI-(B) grown *F. tularensis* LVS were Tx114 phase-partitioned into aqueous (A), detergent (D), and Tx-insoluble phases. The insoluble material was treated with 0.2% sarkosyl to generate soluble (SS) and insoluble (SI) fractions. **A)** Intact fractions derived from 10 µg of WC protein were probed by western blot for the indicated proteins. Predicted locations were derived from the LVS protein sequence with the aid of PSORT (http://psort.hgc.jp/form.html). **B)** Carbohydrates present in proteinase K-treated fractions derived from 10 µg of WC protein were visualized by Emerald Green staining of resolved samples.

To aid our search for differentially-expressed OMPs we fractionated MHB- and BHI-grown *F. tularensis* using Triton X-114 (Tx) phase-partitioning [40], [41], [42], [43] combined with differential membrane solubilization of the Triton-insoluble material. To validate this approach, the resulting fractions along with the parental whole cell lysates (WC) were probed with antisera specific for proteins of known or predicted location. Consistent with previous reports of IglA, IglB, IglC, and GroEL (a chaperone) and KatG being soluble cytoplasmic and periplasmic proteins respectively [31], [44]–[46], we found that these species quantitatively partition into the Tx-aqueous (A) phases (Fig 2A). Curiously, KatG and GroEL along with SodB (superoxide dismutase) and Bfr (a bacterioferritn homolog) have also been found in the culture supernatants of non-host-adapted *F. tularensis* [47], [48]. Active secretion of the bacterial antioxidant enzymes into host cells could limit cellular activation by altering redox-dependent host cell signaling [49]. Given the alterations of KatG and SodB expression we observed in host-adapted *F. tularensis* (Fig 1 and reference [12]), we quantitatively assessed the distribution of KatG, GroEL, SodB, and Bfr in MHB- and BHI-grown cells and cell-free supernatants. To aid the interpretation of our findings, we similarly analyzed the distribution of the *E. coli* α–hemolysin (HlyA), a secreted pore-forming protein [50], [51] whose expression is increased during mammalian infection [52], [53]. As expected for a secreted protein, HlyA was readily detected (96–97% of the total) in the culture supernatants of *E. coli* with only scant levels observed within the bacterial cells (Fig. S1). In stark contrast, the *F. tularensis* proteins were readily detected in bacterial cells with only scant amounts (∼1% of the total) detected in the supernatants and only those of MHB-grown *F. tularensis.* Based on these findings, we conclude that these *F. tularensis* proteins are not actively secreted - consistent with the findings of Hager *et. al.* for LVS [54]. A similar controversy over the active secretion of catalase, chaperones, superoxide dismutase, peroxiredoxins, and others played out in the *Mycobacterium tuberculosis* field in the 1990s including notions of how these bacterial enzymes could modify host cell behavior [55]-[59]. Ultimately, active secretion of these Mtb proteins (and presumably their effector functions within host cells) was unambiguously disproven by Tullius *et. al.* who used approaches similar to those used here to show that the presence of these proteins in cell-free culture supernatants resulted, not from active secretion, but instead from bacterial cell lysis in concert with the high abundance and extra-cellular stability of these proteins [60].

Of the membrane proteins, FsaP contains a single internal, transmembrane domain [61] which would place it in the cytoplasmic membrane [62], [63], a location consistent with both our detection of FsaP in the Tx-detergent phases (which lack the OM markers FopA, TolC, and LPS OAg, Fig 2A) and the inability of polyclonal FsaP-specific Ab to label the surfaces of either intact *F. tularensis* or recombinant *E. coli* expressing FsaP [61]. While contrary to the interpretations of Mellio *et. al.* [61], our assignment of FsaP to the inner membrane is also consistent with the protein's lack of heat-modifiability – a characteristic property of membrane-spanning OM proteins [62] – displayed by FopA but not FsaP (data not shown). Pal (peptidoglycan-associated lipoprotein) and Tul4 (as known as LpnA) are predicted and confirmed lipoproteins respectively [45], [64]. As the rules for lipoprotein sorting in *F. tularensis* have not been established [45], it is not yet possible to predict with confidence the location of these molecules which could be lipid anchored to **i)** the outer leaflet of the inner membrane, **ii)** the inner leaflet of the outer membrane, or **iii)** the outer leaflet of the outer membrane [65]. Pal homologues in gram-negative bacteria are sub-surface moieties anchored by their N-terminal lipids to the inner leaflet of the OM allowing the protein to bind the periplasmic peptidoglycan [65]. Tul4 is a potent TLR2 agonist [66], [67] which is reportedly surface-exposed in capsule-deficient *F. tularensis* but not in the WT bacteria [68]. These cellular locations are consistent with our detection of the lipoproteins primarily in the Tx detergent (D) phases and, to a lesser extent, the Tx-insoluble, sarkysol-soluble (SS) phases (Fig 2A). TolC-B and FopA are well characterized membrane-spanning OMPs [69] and as expected, we detected these proteins in the sarkosyl-insoluble (SI) fraction (Fig 2A).

As a final means of validating our approach, we analyzed these same fractions (after proteinase-K treatment) by SDS-PAGE followed by periodic acid oxidation and fluorescent staining to visualize carbohydrates such as those in LPS - a known OM constituent. As expected, the typical ladder pattern of repeating LPS O-antigen (OAg) units was observed in the WC and SI fractions but not in the A, D, or SS phases (Fig. 2B). However, this carbohydrate staining also revealed unexpected and striking differences between MHB and BHI-grown *F tularensis*.

First, despite equivalent amounts of Lipid A core, the “short” LPS (containing one to three OAg repeats) was more abundant in MHB-grown bacteria whereas the LPS from BHI-grown *F tularensis* was more heavily populated by “long” LPS (see Fig. 2B and Fig. S2 for long and short exposures of the same gel). The increased abundance of “short” LPS in MHB-grown *F. tularensis* was confirmed by western blots (data not shown) with mAb FB11 which is specific for the bacterium's LPS OAg [30]. Another significant difference was apparent in the high-molecular weight (HMW) carbohydrates of MHB- and BHI-grown *F. tularensis*. The MHB WC samples contained a diffuse region of staining extending roughly from ∼100 kDa to slightly above 225 kDa (Fig 2B). In the BHI-grown samples, this region of staining extended well above the 225 kDa marker to nearly the bottom of the loading wells. Upon phase-partitioning, this BHI-specific ≥225 kDa carbohydrate was found in the aqueous phase along with a BHI-specific, protease-resistant, ∼200 kDa putative glycoprotein that is labeled by both carbohydrate- and protein-specific stains (Fig S3). The phase-partitioning behavior of the HMW carbohydrate was similar to that of the *F. tularensis* OAg capsule recently described by Apicella *et. al.* [30]. As both capsules and extended-length LPS molecules can markedly impact the accessibility of OMPs [70], [71], we next sought to determine if host-adaptation of *F. tularensis* alters the production of OAg capsule and/or the accessibility of Ab to OM constituents.

### Host-adapted *F. tularensis* produce more OAg capsule and bind less Ab directed against OM constituents

**Figure 3:**
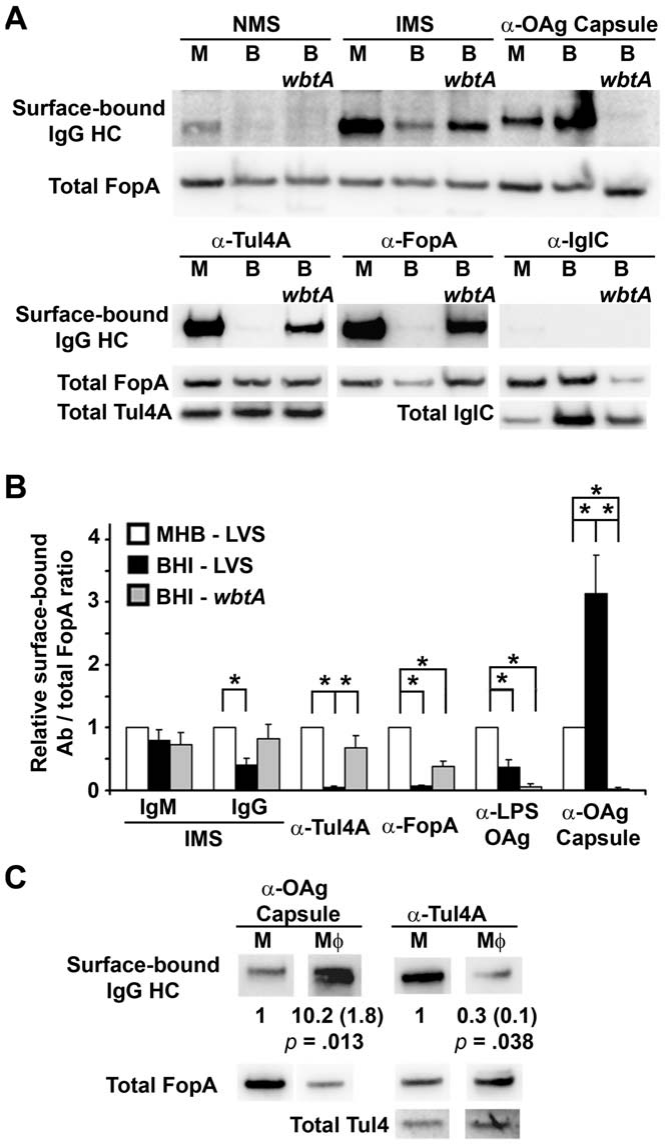
Host-adapted *F. tularensis* produce more OAg capsule and bind less Ab directed against OM constituents. **A)** Intact bacteria (WT and *wbtA*), grown in MHB (M) or BHI (B), were incubated with the indicated sera/Ab, washed and probed for IgG heavy chain by western blot. The probed membranes were stripped and re-probed for total signals for the indicated proteins. **B)** Surface-bound Ab-to-FopA ratios from 5–8 independent experiments were quantified and expressed relative to that of the MHB-grown WT with same Ab. Asterisks indicate values that differed significantly (*p*≤0.05, T-test with Bonferonni correction). **C)** MHB- and MΦ- grown *F. tularensis* were incubated with Ab specific for the OAg-capsule and Tul4 and analyzed as in A above. Mean FopA-normalized, surface Ab values from 3 independent experiments are indicated along with standard deviations in parentheses.

To probe the surface of *F. tularensis* for OAg capsule and OM accessibility we incubated intact bacteria with various Ab and probed the washed bacteria for bound immunoglobulin heavy chain (HC) by western blot. Following development of the Ig HC signals, we re-probed the membranes for total FopA and quantified the data as surface Ab/total FopA and normalized the ratios to the corresponding MHB result. As shown in Fig. 3A and B, when *F. tularensis* was incubated with immune mouse serum (IMS) the amount of IgG bound to the surface of BHI-grown, WT *F. tularensis* was significantly reduced relative to that of its MHB-grown counterpart. The amount of IgG bound to the surface of MHB-grown, WT *F. tularensis* and the BHI-grown, OAg mutant was not significantly different (Fig 3B) and there were no significant differences in the amount of IgM bound to the surfaces of these three bacteria (Fig 3B).

The trend of Ab binding significantly better to the surfaces of MHB-grown *F. tularensis* also was observed with Ab specific for Tul4A, FopA, and LPS OAg (Figs 3A and 3B). This trend, however, was reversed when we probed MHB- and BHI-grown *F. tularensis* with Ab specific for the OAg capsule. BHI-grown *F. tularensis* bound 3-fold more α-OAg capsule Ab than did the same bacterium grown in MHB. MΦ-grown *F. tularensis* also displayed increased production of OAg capsule and reduced OMP (Tul4) accessibility compared to the non-host-adapted bacteria (Fig 3C). As with the BHI-grown bacteria, the reduced Ab binding was not explained by a reduction in Ag expression.

While the increased levels of OAg capsule and reduced accessibly of Ab to OM constituents noted in Fig 3 were internally-consistent, the ∼3-fold increase in OAg capsule observed with BHI-grown *F. tularensis* seemed disproportionate to the 60–95% reduction in binding of α–OMP Ab by the same cells. Additionally, removal of the OAg capsule (in BHI-grown *wbtA*) did not, in all cases, fully restore Ab binding to the levels observed with MHB-grown bacteria. For these two reasons, we postulated that host-adapted *F. tularensis* might produce an additional capsule-like material.

### Host-adapted *F. tularensis* produce an additional high molecular weight carbohydrate that also correlates with reduced accessibility of Ab to OMPs

**Figure 4:**
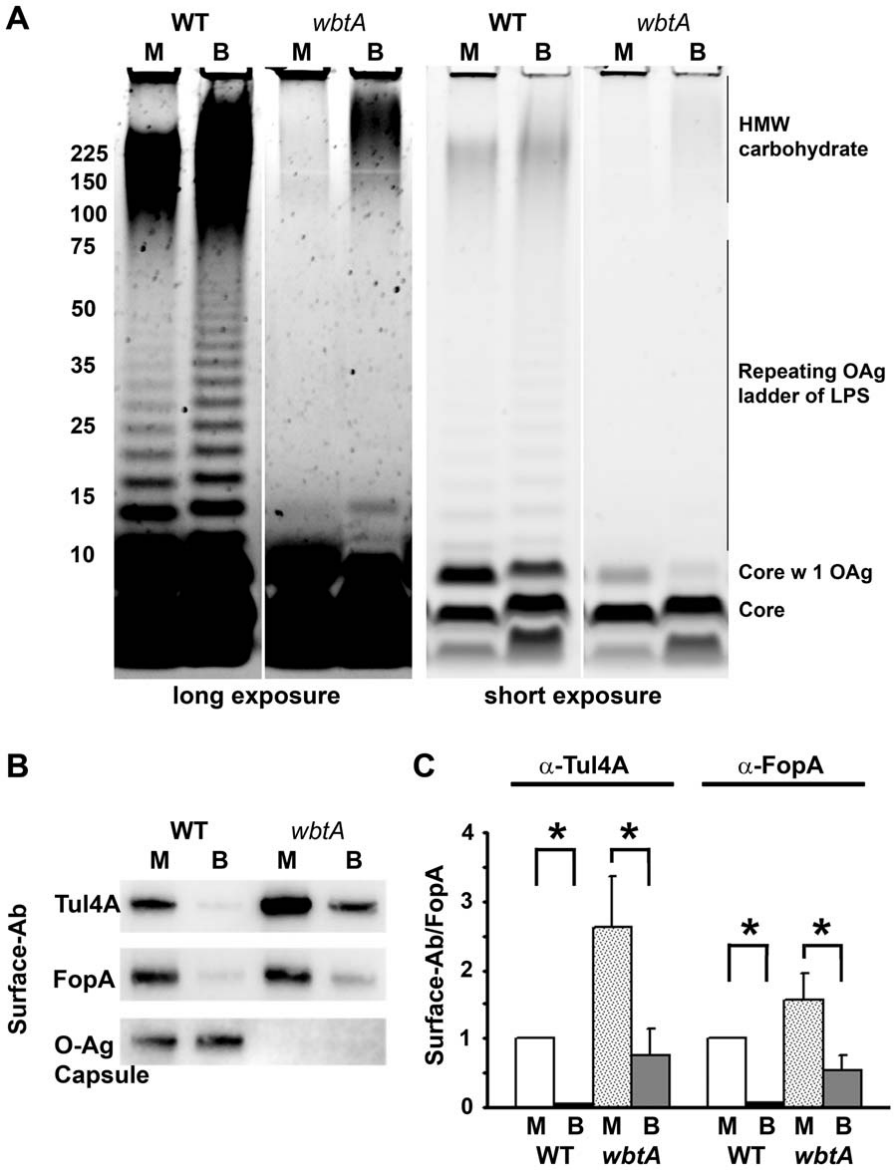
*F. tularensis* grown in BHI produce a HMW carbohydrate, independent of OAg, which correlates with reduced access of OMPs to Ab. **A)** Carbohydrates from the indicated proteinase K-treated samples were visualized as in Fig 2b. **B)** Ab pulldown assays were performed as in Fig 3. **C)** surface-bound Ab to FopA ratios from 3 independent experiments were quantified as in Fig 3. Asterisks indicate values that differed significantly (*p*≤0.05, Bonferonni-corrected T-test).

To determine if growth in BHI induced production of a HMW carbohydrate in addition to OAg capsule we examined the carbohydrate composition of MHB- and BHI-grown *wbtA F. tularensis*. As shown in Fig 4A, the BHI-grown, *wbtA* strain produced far more HMW carbohydrate than its MHB-grown counterpart. This HMW carbohydrate was similar in size (>225 kDa) to the material present in WT, BHI-grown *F. tularensis.* Upon phase-partitioning this material, along with the ∼200 kDa putative glycoprotein, was found in the aqueous phase (Fig S3) similar to what was observed for WT BHI-grown *F. tularensis* (Fig 2B). The aqueous-phase, HMW carbohydrate material derived from BHI-grown, WT *F. tularensis* was slightly more abundant than that of the BHI-grown, *wbtA* mutant (Fig S3) likely indicating that the WT HMW carbohydrate material contains both OAg capsule and the non-OAg HMW carbohydrate.

To determine if the presence of this non-OAg, BHI-specific material correlated with reduced binding of α–OMP Ab, we used MHB- and BHI-grown WT and *wbtA F. tularensis* to immuno-precipitate α-Tul4A and α-FopA Ab as was done for Fig 3. As expected, the *wbtA* strain bound more α–OMP Ab than the similarly grown WT strain. However even in the absence of OAg, BHI-grown *wbtA* bound significantly less surface Ab than its MHB-grown, *wbtA* counterpart (Fig 4B and 4C). This diminished binding could not be explained by differences in loading or Ag expression (data not shown), but could be explained by the BHI-specific, HMW carbohydrate shielding the OMPs from Ab.

### Host-adaptation reduces interaction with effectors of innate immunity

Since the access of many OM constituents to Ab was reduced in host-adapted *F. tularensis,* we postulated that OM constituents might also be shielded from elements of the innate immune system. We tested this notion *in vitro* by examining the ability of differentially-grown bacteria to promote **i)** complement deposition and **ii)** TLR2-dependent cellular activation of naïve MΦs.

**Figure 5:**
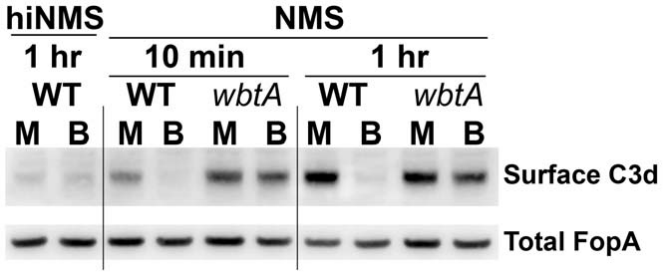
Host-adaptation of *F. tularensis* reduces complement activation. MHB- or BHI-grown bacteria were incubated for 10 min or 1 hr with 25% normal mouse serum (NMS) or heat-inactivated (hi) NMS. Washed bacteria were probed by western blot first with a polyclonal Ab directed against mouse complement protein C3 and then for the *F. tularensis* OMP FopA; the α-C3 Ab was empirically-determined to be selective for the α-chain of murine C3. Results are representative of three independent experiments.

As a respiratory and blood-borne pathogen, *F. tularensis* would be exposed to complement during the bacterium's extra-cellular phase within mammalian hosts [72]. Since LPS- and/or capsule-mutants of *F. tularensis* fix complement more readily than WT bacteria [73]–[77], we hypothesized that the regulated LPS and capsule changes that are commensurate with host-adaptation would diminish complement deposition. As shown in Fig 5, MHB-grown *F. tularensis* exposed to complement-active normal mouse serum rapidly accumulated a ∼35 kDa C3 fragment (consistent with the size of C3d) that failed to accumulate on BHI-grown, WT bacteria. Fragments consistent with the sizes of the α-chain in C3b (∼100 kDa), iC3b_1_ and iC3b_2_ (∼68 and ∼62 kDa) followed a similar pattern of deposition, albeit with different kinetics (Fig S4). Detection of these fragments is consistent with the reported ability of *F. tularensis* to bind factor H [78] which, in conjunction with Factor I, cleaves C3b [79]. As expected [73], deposition of complement was accelerated by the absence of OAg (*wbtA* strains). However, the BHI-grown *wbtA* strain still accumulated less C3d than MHB grown *wbtA* – indicating that the non-OAg, HMW carbohydrate produced by BHI-grown bacteria may also impede complement deposition. At this time we do not know which pathway(s) (classical, alternative, mannose-binding lectin) were active in our assays.

One of the most thoroughly characterized innate cellular responses to *F. tularensis* is the TLR2-dependent activation of naïve MΦ resulting in the secretion of T_H_1 pro-inflammatory cytokines such as TNF-α and IL1-β [80]-[83]. Since bacterial lipoproteins (such as Tul4 - a canonical *F. tularensis* lipoprotein) are thought to be the principal agonists for TLR2-mediated activation [66], [67], [84], our observation that host-adaptation shields Tul4 from Ab suggested that *Ft* lipoproteins might also be shielded from interaction with TLR2. To test this hypothesis, we incubated naïve MΦ with *F. tularensis* that, through either genetic lesion or different growth conditions, had differing levels of HMW carbohydrates and distinct Ab accessibilities to Tul4.

**Figure 6:**
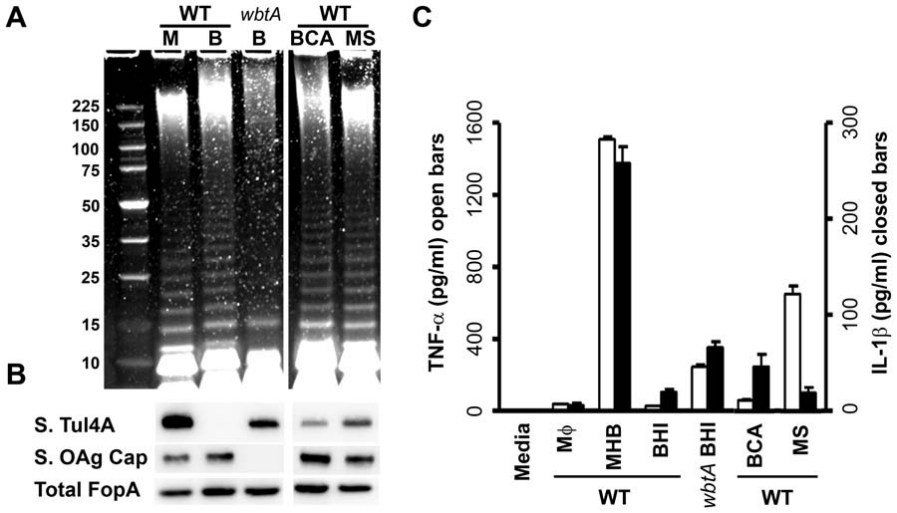
*F. tularensis* surface carbohydrates impact the accessibility of TLR2 ligands and the extent of TLR2-dependent macrophage activation. *F. tularensis* LVS WT and *wbtA* strains were grown in MHB (M), BHI (B), MHB supplemented with spermine (MS), or BHI supplemented with casamino acids (BCA). A) Bacterial lysates containing 10 µg of protein were treated with proteinase K prior to SDS-PAGE resolution and Emerald Green staining of carbohydrates. B) Intact bacteria were incubated with anti-Tul4A Ab, washed and probed for IgG heavy chain by western blot to detect surface-bound (S.) α–Tul4A Ab. The probed membranes were stripped and re-probed for the total FopA and total Tul4. C) Viable *F. tularensis* were incubated with C57BL/6 BMDMs at an MOI of 100 for 24 hrs prior to analysis of secreted cytokine levels shown as means and standard errors.

Compared to MHB-grown *F. tularensis*, bacteria grown in BHI or MΦ (both of which have more capsule and less Ab-accessible Tul4 [Figs 3, 6A+B]) elicited significantly less TNF-α and IL-1β from bone-marrow derived macrophages (BMDM) (Fig 6C, *p*<0.001 for both TNF-α and IL-1β). In the absence of OAg, Tul4 is more surface-accessible and BMDMs responding to OAg-deficient bacteria produced significantly more TNF-α and IL-1β (BHI WT and *wbtA*, *p*<0.001 for TNF-α and *p*<0.05 for IL-1β Fig 6). Supplementation of BHI with casamino acids (BCA) to the level found in MHB suppresses many facets of host-adaptation (Fig 1 and [12]). Here we found that bacteria grown in BCA have reduced levels of HMW carbohydrate and increased surface-accessibility of Tul4 compared to their BHI-grown counterparts (Figs 6A and 6B). Paradoxically, BCA grown bacteria bound more α-OAg capsule Ab suggesting that the observed reduction of HMW carbohydrate primarily reflects decreased production of the non-OAg, putative capsule. The BCA-grown WT *F. tularensis* also provoked significantly higher levels of TNF-α and IL-1β from BMDMs (Fig 6C, *p*<0.028 for both TNF-α and IL-1β). The addition of spermine to MHB (MS) has been shown by Carlson *et al* to increase transcription of *wbtA* by *F. tularensis* and to decrease TNF-α production by MΦ responding to MS-grown *F. tularensis* LVS and SchuS4 [34]. We found that MS-grown *F. tularensis* have slightly longer LPS-OAg, slightly increased levels of HMW carbohydrate, increased binding of α-OAg capsule Ab, and reduced accessibility of Tul4 (Fig 6A+B) compared to MHB-grown bacteria. MS-grown bacteria also evoke significantly less TNF-α and IL-1β from BMDMs (*p*<0.001 for both TNF-α and IL-1β in Figs 6C).

### Host-adaptation accelerates tularemia pathogenesis

Previously we reported that mice challenged with BHI-grown *F. tularensis* LVS reproducibly succumb to infection ∼1 day sooner than mice challenged with an equivalent dose of MHB-grown bacteria [12]. At the time, we assumed that this difference was largely an indication that the host-adapted bacteria were better prepared for the metabolic/nutritional environment of the mammalian host. In light of the LPS and capsule changes noted above, it is likely that the differences in the survival also reflected the heightened susceptibility of non-adapted bacteria to host innate immune effectors that could target the bacterium for clearance before it could host-adapt. Here we sought to extend our findings with LVS to the human-virulent strain *F. tularensis* SchuS4.

**Figure 7:**
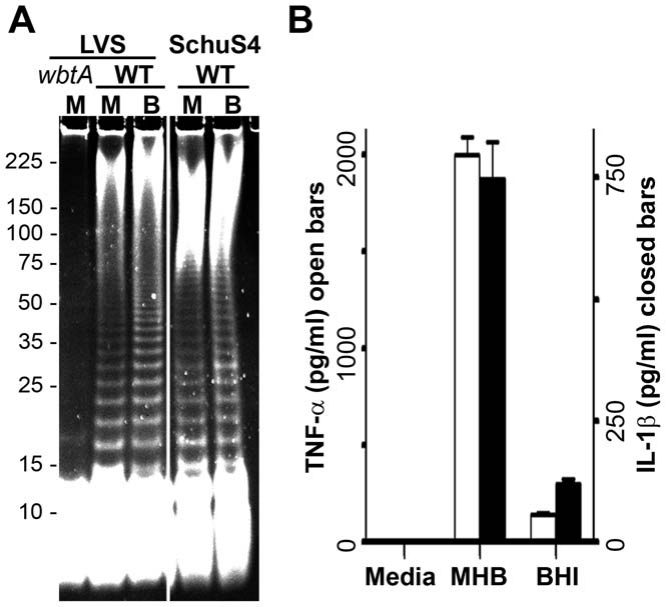
BHI-grown *F. tularensis* SchuS4 also displays increased production of HMW carbohydrate(s) and reduced cytokine elaboration. *F. tularensis* LVS (WT and *wbtA*) and SchuS4 were grown in MHB (M) and BHI (B). A) Proteinase K-treated lysates were resolved through 8-16 % SDS-PAGE Protean II large gels and stained with Emerald green to visualize carbohydrates. B) Viable *F. tularensis* SchuS4, grown as indicated, were co-incubated with C57BL/6 BMDMs at an MOI of 100 for 24 hrs prior to analysis of secreted cytokine levels. Results are means and standard errors from three independent experiments each conducted in duplicate.

We have previously found protein expression by differentially-grown SchuS4 to follow a pattern similar to that of differentially-grown LVS [12]. Accordingly, here we examined the carbohydrate composition of differentially-grown SchuS4. As shown in Fig. 7A, two features of BHI-grown LVS are conserved in BHI-grown SchuS4 – **i)** an increase in the proportion of LPS molecules bearing a large number of OAg-repeating units, and **ii)** an increase in the size of the HMW carbohydrate. Interestingly, SchuS4 appears to produce more HMW carbohydrate than LVS. Next we examined TNF-α and IL-1β production by BMDM responding to differentially-grown SchuS4. As shown in Fig 7B and similar to our observations with LVS, BHI-grown SchuS4 elicited significantly lower levels of these pro-inflammatory cytokines from murine BMDMs and human U937 cells (data not shown) compared with that provoked by MHB-grown SchuS4 (*p*<0.001 for both TNF-α and IL-1β from BMDMs).

Finally, we examined survival of C57BL/6 mice challenged with MHB- or BHI-grown, *F. tularensis* SchuS4. In our experience, SchuS4 grows slightly faster than LVS suggesting that these bacteria might host-adapt more quickly than LVS and that differences in survival of SchuS4-infected animals might be less than was previously observed with LVS [12]. Consistent with the above predictions and the presumably heightened susceptibility of non-adapted bacteria to the restrictions present in the host environment (nutritional, innate immune, *etc*), we observed (in 3 independent experiments initiated with distinct preparations of bacteria) that lethality among mice challenged with MHB-grown SchuS4 began later and took longer to complete than in mice challenged with BHI-grown bacteria (data not shown). For the mice infected with BHI-grown SchuS4, mortality began 5 days (120 hrs) post-infection (PI) with a median survival time of 132 hrs. By day 6 (144 hrs) PI, all mice had succumb to infection with BHI-grown bacteria. For the mice challenged with MHB-grown SchuS4, mortality began at 126 hrs (median survival time  = 144 hrs) but was not complete until day 7.25 (174 hrs) PI. The differences in survival of mice infected with MHB-grown and BHI-grown *F. tularensis* SchuS4 were highly-significant (*p*<0.001).

## Discussion

We began these studies with the goal of identifying Ab-accessible OMPs expressed by host-adapted *F. tularensis;* these proteins could then be evaluated as subunit vaccine candidates. Pursuit of this goal led us to the discovery that host adaptation includes multiple changes to the bacterium's surface carbohydrates (LPS and capsules) which cumulatively hinder Ab, complement, and TLR2-mediated recognition of OM constituents.

Within the mammalian host, *F. tularensis* undergoes intracellular replication yet has a significant extracellular phase [5], [7] which likely facilitates dissemination. During this latter phase *F. tularensis* is presumably exposed to a variety of anti-bacterial factors such as complement, antibody, and phagocytes - which if activated – can hinder the bacterium's progress [85]. For many pathogens, significant protection is afforded by a large polymeric capsule that surrounds the bacterium and limits access of immune effectors to the bacterium's limiting membrane [86]–[88]. In this regard, it is not surprising that *F. tularensis* also would utilize this highly successful strategy. However, the existence and/or composition of *F. tularensis* capsule(s) have remained a subject of controversy.

**Figure 8:**
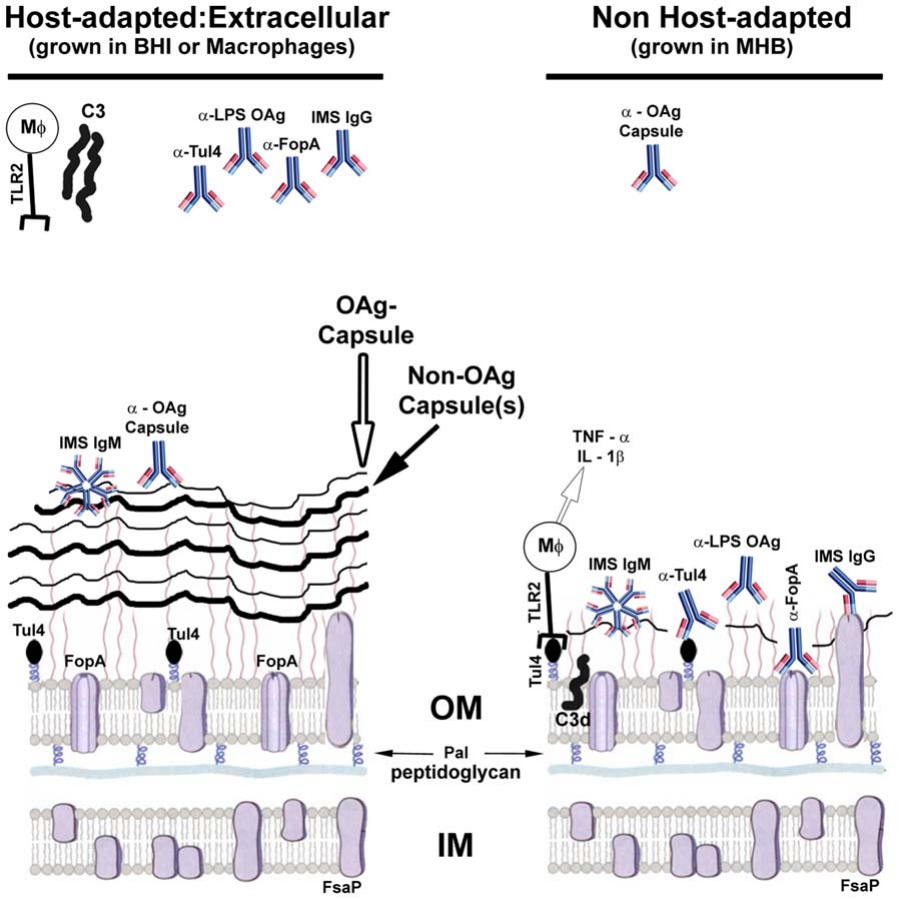
A model of the interaction of MHB-grown and host-adapted *F. tularensis* with select mammalian immune effectors. The cellular locations depicted for FopA, Tul4, Pal, and FsaP are based upon data in figure 2A and the relevant text in which this data is discussed. The depiction of host-adapted bacteria having longer LPS O-Ag carbohydrate chains and elevated levels of capsular material is based upon results in figures 2B, 3, 4, 6A, 7A, S2, and S3. The depiction of antibodies binding differentially to host-adapted and non-adapted bacteria is based on results presented in figures 3, 4, and 6. The differential binding/activation of complement protein C3 is based upon data in figures 5 and S4. Our model of TLR2-dependent production of T_H1_-type pro-inflammatory cytokines by macrophages responding to *F. tularensis* is based upon results presented in figures 6B and 7B.

Several electron microscopic (EM) analyses have noted circumferential labeling of bacteria that had been negatively-stained with heavy metals or immuno-labeled with α-*F. tularensis* sera - thereby providing support for the existence of an *F. tularensis* capsule [89], [90], [76]. In contrast, several recent attempts to detect a capsular-structure and/or material have been unsuccessful [73], [91], [92], prompting speculation that LPS OAg might be the only capsule-like structure produced by the bacterium [92]. Initial clues to the resolution of this conceptual discord were provided by the observation that repeated serial passage of *F. tularensis* in a chemically-defined, synthetic medium increased the abundance of an EM-visible capsule-like material [93]. This finding suggested that capsule production by *F. tularensis* might be environmentally regulated, however, it cannot be excluded that extended passage led to accumulation of encapsulated variants. Recently Apicella *et. al.* provided another advancement by generating a capsule-specific reagent (mAb 11B7) and utilizing SDS-PAGE to visualize the bacterium's HMW carbohydrates [30]. These tools, along with biophysical and immunological approaches, were employed to determine that *F. tularensis* produces an OAg capsule that is physically distinct from that of the LPS OAg. Here we synthesized these multiple observations and experimental approaches and found that host-adaptation of *F. tularensis* increases production of **i)** OAg capsule, **ii)** longer polymers of LPS OAg, and **iii)** an additional HMW carbohydrate/glycoprotein material(s) with capsule-like functions. We speculate that the putative glycoprotein described here may be related to the capsule-like complex, a putative glycoprotein, recently characterized by Bandara et al [94]. Collectively these changes shield OM constituents from recognition by Ab, complement, and TLR2-dependent macrophage activation – functions of capsules and/or long LPS which have been well-characterized in other pathogens [70], [71], [87], [95]–[97] which are now beginning to be explored in *F. tularensis* [73], [75]. Our working model for the interactions of differentially-grown *F. tularensis* with select immune effectors is presented in Figure 8.

Our results provide both some confirmatory observations and several novel findings. Perhaps most striking is the marked induction of multiple capsular-materials and functions that is commensurate with host-adaptation of *F. tularensis* (observed with bacteria grown in MΦ or BHI). Host-adapted bacteria bound significantly higher levels of the mAb 11B7 (which labels OAg-capsule) and contained additional, non-OAg, HMW carbohydrates absent in MHB-grown bacteria. The elevated production of capsule/HMW carbohydrates along with the lengthening of the LPS OAg polymers correlated with a profound (60-95%) reduction in the binding of multiple Ab that have reactivity with OM constituents. These Ab include the polyclonal (p) IgG (but not IgM) portion of immune sera (raised through sequential infections with LVS followed by SchuS4) as well as pAb and/or mAb specific for the lipoprotein Tul4A, the integral OMP FopA, and the LPS OAg. Interpreting the reduced binding of IMS IgG by host-adapted *F. tularensis* is complex in that this sera has a broad range of specificities that likely include capsule(s) as well as Tul4, FopA, LPS OAg and many others. However, since Tul4, FopA and LPS are immuno-dominant Ags [29], [32], [64], and capsule is reported to be poorly-immunogenic [90], our observation (that host-adapted [capsule-expressing] bacteria bind less IMS IgG) is consistent with an increased amount of poorly-immunogenic capsule blocking access of Ab to the more highly immunogenic OM moieties. The use of WT and OAg-mutant bacteria allowed us to conclude that both the OAg and non-OAg HMW carbohydrates inhibit Ab binding. However, based on our findings, we can not dissect the relative contributions of **i)** the OAg capsule and **ii)** the longer LPS OAg polymers to the shielding of OM moieties from Ab.

The results with the mono-specific Abs are most readily interpreted as an indication that elevated capsule production limits access of Ab to Ag. Alternatively, host-adapted bacteria could have increased production (or acquisition) of a protease that cleaves bound Ab. Indeed *F. tularensis* has been reported to bind exogenously-added, active plasmin [98], [99] which, in one report, cleaved both bound, opsonizing IgG and unbound, non-specific IgG [99]. For several reasons (in addition to the fact that we did not add plasmin) we conclude that proteolysis of Ab is not the mechanism underlying the differences we observed. First, while the amount of bound IgG specific for OM constituents (α-Tul4, α-FopA, α-LPS OAg) was reduced in host-adapted bacteria, the amount of surface-bound IMS-IgM was unchanged and the amount of bound IgG specific for another constituent (α-OAg capsule) was increased. Secondly, the reduction in binding of α–OMP Ab (α-Tul4, α-FopA) by BHI-grown bacteria was largely negated in bacteria (*wbtA*) unable to produce intact LPS and OAg capsule. Lastly, the addition of protease inhibitors to BHI-grown bacteria did not increase the amount of bound Ab (data not shown). Accordingly, we conclude that the elevated capsule production by host-adapted *F. tularensis* limits the access of Ab to multiple OM antigens.

While both **i)** complement activation (and subsequent deposition), and **ii)** TLR2 engagement (and subsequent cytokine production), are more complex processes than Ab binding, we postulate that the increased capsule present on host-adapted *F. tularensis* is also limiting access of these innate immunity molecules to bacterial OM ligands. Activation of C3 leads to deposition of C3b that contains an α-chain of ∼100 kDa which is covalently-bound to an acceptor molecule on the bacterial surface. The cooperative activities of Factors H and I cleave C3b sequentially to yield α–chain fragments (iC3b_1_, iC3b_2_, and C3dg/C3d) of ∼68, ∼62, and ∼40/∼35 kDa; these C3b fragments retain the ability to be bound by complement receptors but do not further promote the complement cascade [79], [100]. In our assays with normal mouse sera as the source of complement, we observed the above cascades from activation to C3d (or C3dg) production. However, this was only observed with MHB-grown WT bacteria and OAg-deficient *F. tularensis*. In contrast, host-adapted WT bacteria showed no evidence of C3 deposition, and BHI-grown, OAg-deficient bacteria accumulated less C3d/C3dg than their MHB-grown counter parts. The simplest (although not exclusive) interpretation of these findings is that the C3-activating moieties (natural Ab, mannose-binding protein, or targets of the alternative pathway) are shielded from their ligands(s) by capsule similar to what we hypothesize for the reduced binding of OM-specific Ab to host-adapted *F. tularensis*. It is conceivable that the heightened sensitivity of MHB-grown bacteria to complement may have contributed to the more rapid demise of mice infected with BHI-grown *F. tularensis* that we report here for strain SchuS4 and observed previously with strain LVS [12].

The activation of naïve MΦs in vitro by MHB-grown bacteria has been shown by multiple groups to be TLR2-dependent and to characteristically culminate in the elaboration of T_H_1-type pro-inflammatory cytokines [80]-[83]. However, these cytokines are largely undetectable in the lungs of mice during early respiratory tularemia (days 0 to ∼3) even though this is a time and site of exponential bacterial replication. This apparent discord has prompted the notion that the bacterium is actively suppressing the inflammatory response. The active-suppression hypothesis is consistent with the observations of many that *F. tularensis*-infected cells and/or animals show blunted T_H_1-type pro-inflammatory responses to exogenous TLR agonists [101]–[104]. Active-suppression of host-cell responses by bacteria is typically mediated by bacterial effector proteins which are delivered to the host cell cytoplasm by either a bacterial syringe-like apparatus (T3SS and T4SS) or as the cargo components of AB-toxins. *F. tularensis* is notable for its non-proinflammatory LPS, its lack of identifiable toxins and its lack of T3SS and T4SS syringe-like apparatuses [105]; a related species *F. novocida* may have a functional T6SS [106].

Our findings - that host-adapted bacteria produce sufficient surface carbohydrates to shield known TLR2 ligands (such as Tul4A) from Ab, and apparently from TLR2, - suggest a distinct mechanism to explain the apparent hypo-responsiveness of MΦs engaging *F. tularensis*. Specifically we postulate that host-adapted bacteria, through shielding of TLR2 ligands with abundant capsular carbohydrates, either **i)** simply fail to trigger the TLR2 signaling cascade that results in production of TNF-α and IL-1β, and/or **ii)** preferentially engage a distinct receptor that provokes an non-T_H_1 type response. Along these lines, the mannose-receptor, which has been implicated in the phagocytosis of *F. tularensis* by MΦs and suggested to be a “safe portal of entry” [107], [108], is an attractive candidate as pathogen-mediated ligation of this receptor can blunt TLR-driven T_H_1-type pro-inflammatory responses [109]. We envision that the mannose receptor could be preferentially engaged by the abundant HMW carbohydrate capsule present on host-adapted bacteria. An early analysis of total capsular material (presumably containing both OAg capsule and non-OAg capsule) detected high levels of mannose [90] while a recent analysis of purified OAg capsule revealed an absence of mannose [30]. These observations would be consistent with the non-OAg capsule (enriched on host-adapted bacteria) being the presumptive ligand of the mannose receptor. It is conceivable that earlier and/or more extensive engagement of the mannose receptor, combined with reduced TLR2 engagement, may have contributed to the more rapid demise we observed with mice infected with BHI-grown SchuS4.

While we used host-adapted and non-host-adapted bacteria primarily as tools to explore the impacts of capsule production on immune effector functions, we believe that our results also have implications for understanding the regulation of capsular components during the host-cell infection cycle. Within the cytoplasm of mammalian host cells the concentrations of both free amino acids and spermine are high compared to those of the extracellular environment. We have shown previously [12], and confirmed here, that BHI-grown *F. tularensis* and extracellular, host-adapted bacteria (those which have emerged from infected MΦ) are phenotypically indistinguishable; much of this MglA-dependent phenotype is repressible by high concentrations of free amino acids. Using microarrays, RT-PCR, and transcriptional reporters to compare *F. tularensis* grown in MHB and MS or harvested from the cytoplasm of infected cells, Carlson et al [34] found that MS-grown and intracellular bacteria are highly similar; spermine activates promoters within multiple insertion sequences including one upstream of *wbtA* – thought to be involved with the production of OAg. Here we found that, compared to bacteria grown in MHB, MS-grown *F. tularensis* have increased binding of mAb specific for the OAg-capsule (shown in Fig 6). We also found that production of OAg capsule was also stimulated by high concentrations of free amino acids. The responsiveness of OAg capsule to spermine and amino acids suggests that the OAg capsule is important, not only for extracellular survival, but also for intracellular growth. Indeed Lindemann *et. al.* have recently shown that mutants with defects in production and/or export of OAg capsule, in addition to being complement sensitive, have impaired cytosolic replication despite showing normal phagosome-escape [75].

In contrast to the OAg capsule, we believe that one-or-more of the non-OAg capsular materials is repressed by high concentrations of free amino acids possibly suggesting a primarily extracellular role for this material. We found that addition of casamino acids to BHI decreased the production of HMW carbohydrate by *F. tularensis* and that this decrease was not attributable to changes in OAg (shown in Fig 6). We speculate that this amino-acid inhibitable, extracellular HMW carbohydrate(s) that we observed maybe similar to the putative capsule noted by Golovliov *et. al.* [89]. This material was abundant on extracellular bacteria yet markedly reduced/absent on intracellular bacteria. Down-modulation of this non-OAg capsular component once inside the host cell could facilitate more rapid host-pathogen interactions such as nutrient acquisition. In the extracellular milieu this capsular component could impede recognition by complement, Ab, and TLR2. We postulate that the higher level of capsule (OAg and non-OAg) present on BHI-grown *F. tularensis* may have contributed to the more rapid demise we observed of mice infected with BHI-grown SchuS4 (this work) and LVS [12].

The survival times we observed for C57Bl/6 mice challenged with ∼20 CFU of MHB-grown SchuS4 (median survival time - 6 days) are highly similar that those recently reported by Ireland *et al* in which Balb/c mice were challenged with 25 CFU of MHB-grown SchuS4 (mean time-to-death of 5.8 and 5.4 days in separate experiments) [110]. On the other hand, the statistically significant differences we observed between mice challenged with MHB- and BHI-grown SchuS4 (median survival time - 12 hr difference, time-to-complete lethality - 30 hr difference) are similar to differences recently reported by groups examining the virulence of genetically distinct *F. tularensis* type A strains. Molins et al monitored survival of mice challenged with different clades of type A strains and observed mean time-to-death differences of 9 hr (between A1a strains and A2 strains) and 13.5 hrs (between A1a strains and A1b strains) [111]. Other groups have examined survival of mice challenged with WT type A strains and isogenic mutants (*pilQ*, *tolC*, *rep*, *hfq*, and FTT0609) and found differences in time-to-complete lethality of 24 hrs [112], [113] – similar to the 30 hr difference we report here for host-adapted vs non adapted SchuS4. Thus the impact of host-adaptation on murine lethality experiments is similar in magnitude to the bacterial genetic differences mentioned above.

In addition to the above experiments with mice lacking pre-existing specific immunity; we predict that the use of host-adapted or non-adapted challenge inoculums could also impact the outcome of vaccine efficacy trials. This could be especially true in the case of OMP-based, sub-unit vaccines - where the accessibility of the OMPs to Ab differs depending on how the challenge inoculm was prepared (consider Fig 8). In this case, vaccine-induced α–OMP Ab (through recruitment of phagocytes and/or complement) could clear a portion of the challenge inoculm before the bacteria had host-adapted; after host-adaptation these Ab would have reduced/no efficacy. In total, such an experiment could report the vaccine to be “partially protective” when in fact this might be true only for a MHB-grown challenge dose. Infection with *F. tularensis* emanating from a tick, tabanid fly, mosquito, aerosolized lagomorph, or the “lab” of a terrorist that does not use MHB could proceed unhindered in the face of such vaccine-induced immunity. Accordingly, we suggest that vaccine discovery and testing should incorporate the use of host-adapted *F. tularensis* to avoid re-trekking the OspA path.

## Materials and Methods

### Bacteria and Media

Wild-type (WT) *F. tularensis* LVS and the isogenic mutant strains *mglA*, *pmrA*, and *wbtA* have been described previously [12], [36], [114]. *F. tularensis* SchuS4, originally isolated from a human case of tularemia, was obtained from the U.S. Army Medical Research Institute for Infectious Diseases (Frederick, MD). All experiments using SchuS4 were conducted within the Albany Medical College ABSL-3/BSL-3 facility which has been certified by the Center for Disease Control. SchuS4 lysates for protein analysis to be performed outside of this facility were generated by boiling harvested bacteria in 50 mM Tris, pH 8.0 containing 1 % SDS followed by sterility testing. *Escherichia coli* WAM 1824, which secretes α-hemolysin (HylA), has been described [50].

Routine culturing of *F. tularensis* involved streaking aliquots of frozen bacterial glycerol stocks onto Mueller Hinton Chocolate agar plates [Becton, Dickinson and Company (BD), Sparks, MD] followed by 2–3 days of growth in a humidified chamber maintained at 37°C. Starter cultures were generated by resuspending several isolated colonies in ∼100 µl of BHI from which ∼50 µl was immediately used to inoculate 3–5 mls of BHI and MHB for ∼18 hrs of growth on an orbital shaker operating at 200 rpm and 37°C. Mature starter cultures were used at a 1∶100 dilution to inoculate larger volumes (10–50 ml within 125–250 ml Erlenmeyer flasks) of fresh media appropriate for each experiment. Unless stated otherwise, all bacterial cultures used in this work were in mid-log phase (OD_600_ = 0.4–0.6). Preparation of MHB and BHI media as well as supplementation of BHI with casamino acids has been described previously [12]. Supplementation of MHB with spermine to 200 µM was performed as described [34].

### Tissue Culture

Unless stated otherwise, tissue culture was performed using high-glucose DMEM (HyClone. Logan, Utah) containing 10% fetal bovine serum (Pel-Freez. Rogers, AK), 100 U/ml penicillin/100 µg/ml streptomycin (Gibco/Invitrogen. Grand Island, NY), 2 mM GlutaMAX (Gibco), 10 mM HEPES (Mediatech, Inc. Herdon VA), 0.075 % sodium bicarbonate (Mediatech), and 50 µM β-mercaptoethanol (Sigma). Bone-marrow-derived macrophages (BMDM) were harvested from C57/BL6 mice (Taconic Farms, NY); the murine macrophage-like cell line RAW 264.7 was cultured as described [33]. Murine hybridomas producing mAb directed against IglB and IglC were graciously provided by Francis Nano (U. of Victoria).

### Generation of macrophage-grown bacteria

To generate macrophage (MΦ)-grown *F. tularensis* LVS, adherent RAW cells at ∼75 % confluence within four-to-six T-75 flasks were rinsed and replenished with antibiotic-free DMEM media (13 ml/flask). MHB-grown bacteria were harvested by centrifugation (4000 x *g*, 10 min) and resuspended in 1 ml of antibiotic-free DMEM media. MΦ infections were initiated at an MOI of 500 and allowed to proceed for 8 hrs within a tissue-culture incubator. Monolayers were rinsed and incubated for 1 hr with DMEM containing 50 µg/ml gentamicin. Following five further rinses, the cultures were replenished with fresh antibiotic-free DMEM prior to further incubation. At 36 hrs post-infection, the extracellular bacteria present in the tissue-culture supernatant were subject to 2–4 low-speed (500 x *g*, 10 min) spins to remove any MΦ; supernatants from each spin were examined by darkfield microscopy for contaminating eukaryotic cells. The bacteria present in the MΦ-free supernatants were harvested by centrifugation (7000 x *g*, 10 min) and gently resuspended to ∼5×10^9^/ml in antibiotic-free DMEM.

### SDS-PAGE and western blot analysis

Samples of *Francisella* [10 µg (∼1×10^8^ cells)] were mixed with Laemelli sample buffer and boiled for 10 min prior to resolution through 4–12 % gradient SDS-PAGE pre-cast gels (Invitrogen). The running buffer was NuPAGE MES SDS buffer from Invitrogen; gels were variously run at 90–160 V. Resolved gels were stained with either coomassie blue (BioRad) or transferred to nitrocellulose membranes. Coomassie-stained gels were scanned into Adobe Photoshop using an HP 2820. Membranes to be probed with mAb were blocked for 15 min with PBS, 0.05 % Tween 20, 1% casein; blots to be probed with polyclonal sera were blocked for 1 hr with PBS, 0.05% Tween 20, 2.5% horse serum, 1% casein. Polyclonal sera were applied for 1–2 hr at dilutions ranging from 1∶1000 to 1∶60,000. Supernatants from mAb-producing hybridomas were applied for overnight (o/n) incubations. The mAb FB11 specific for *F. tularensis* O-antigen (Abcam) was used o/n at a dilution of 1∶1000. Blots to be probed multiple times were first probed with mAb prior to stripping and reprobing with polyclonal sera. HRP-conjugated secondary antibodies were used at dilutions ranging from 1∶1000 to 1∶20,000. Development of the chemiluminescent substrate (SuperSignal West Pico, Pierce, Rockford, IL) was visualized using an Alpha Innotech imaging system in movie mode. Densitometric analysis of developed blots was performed on the same system.

### Antibodies

Primary antibodies were generously provided by Francis E. Nano (University of Victoria) and Terry Otto (Immuno-Precise Antibodies, Ltd., α-MglB, α-IglC, α-IglB); Daniel L. Clemens (University of California Los Angeles, α-GroEL, α-KatG, α-SodB, and α-Bfr); Micheal V. Norgard and Jason F. Huntley (University of Texas Southwestern Medical Center, α-Tul4A, α-Mip, α-Pal, and α-FopA); Eric R. LaFontaine (University of Georgia, α-FspA); Jorge Benach, Martha Furie, and Anne Savitt (SUNY Stony Brook, mAb α-Tul4A/LpnA and α-FopA); and Michael Apicella (University of Iowa, mAb 11B7 α-OAg capsule). Normal mouse serum (NMS) was collected from naïve C57/BL6 mice and prepared in a manner to preserve complement activity. Rabbit polyclonal sera against the secreted *E. coli* protein HlyA was provided by Rodney Welch (University of Wisconsin-Madison). To generate infection-derived immune mouse sera (IMS) C57/BL6 mice were sequentially infected with 1000 CFU of LVS followed 14 days later by 20 CFU of SchuS4. IMS was harvested 11 days post SchuS4 infection, filtered (0.22 µm) and tested for sterility prior to use. The mAb FB11 specific for *F. tularensis* O-antigen was purchased from Abcam. Biotinylated goat α-Ig heavy (γ) chains of mouse, rat, and rabbit IgG or IgM were from SouthernBiotech (Birmingham Al) and used in western blots O/N at a dilution of 1∶1,000; these primary Ab were detected using strepavidin-conjugated HRP. FITC conjugated Goat α–mouse C3 was purchased from Immunology Consultants Laboratory. By comparing the reactivity of this Ab in western blots against whole NMS prepared in the presence and absence of β–ME we found that this Ab to be selective for the α-chain of C3.

### Fractionation of *Francisella*

Mid-log phase *F. tularensis* was harvested by centrifugation (8000 x *g*, 15 min, 20°C). Supernatants were decanted and sterile (0.22 µm)-filtered for subsequent analysis; the bacterial pellet was resuspended in fresh growth media (MHB or BHI), transferred to pre-weighed Eppendorf tubes, pelleted as above after which the transfer supernatant was aspirated. Pellet wet weights were determined and used to estimate cell numbers based upon the estimate of 1 mg of wet weight  = 5×10^8^ bacteria. Cells were resuspended to 2.5×10^7^/µl (higher bacterial concentrations promoted protein precipitation in subsequent steps) in 20 mM Tris, pH = 8.0 containing 100 mM NaCl, 20 µl/ml protease inhibitor cocktail (Sigma, #P8849). After determining the protein concentrations (DC protein assay, BioRad, Hercules, CA), equilibrated bacterial suspensions were supplemented with EDTA to 5 mM, lysozyme and RNAse to 100 µg/ml each, Benzonase (1 µl/ml, Sigma), and Triton X-114 (Acros, NJ) to 1% from a 10% stock in PBS. Following a 1 hr incubation at room temperature with periodic gentle agitation, a 1/20 vol. aliquot was saved as the whole-cell (WC) fraction; the WC and remaining sample were stored at −20°C overnight. The samples were thawed and incubated at RT until further viscosity reduction abated. Following 15 min incubation on ice, the samples were centrifuged (20 min, 16,500 x *g*, 4°C) to yield Triton X-114 soluble (TxS) and insoluble (TxI) fractions. The TxS fractions were transferred to fresh tubes and centrifuged again to remove any TxI carry-over; the TxI fractions were washed twice with cold PBS (1 vol). The TxS fractions were incubated in a 32°C water bath for ∼10 min followed by centrifugation (7000 x *g*, RT, 15 min) to effect phase partitioning [40]–[43], [64] into aqueous (top) and detergent (bottom) phases (any protein precipitation at this step rendered the samples useless, such experiments were discarded). Recovered phases were washed by supplementation (to 1% Triton X-114 - A or 10 vol PBS - D) followed by phase separation; three washes per recovered phase were performed. The washed Triton insoluble (TxI) phases were resuspended in PBS containing 0.2% sarkosyl (Fisher Scientific, Fair Lawn, NJ) and incubated for 20 min with gentle agitation followed by centrifugation (30 min, 4°C, 16,500 x *g*). The sarkosyl-soluble (SS) fraction was recovered and centrifuged a second time to remove any carryover of insoluble material. The sarkosyl-insoluble (SI) material was washed once with PBS, 0.2% sarkosyl and dissolved in 10 mM Tris, pH = 8.0 containing 1 % SDS. WC, A, D, SS, and SI fractions were resolved by SDS-PAGE.

### Emerald Green staining of SDS-PAGE gels

Boiled samples of *F. tularensis* in Laemelli sample buffer (1 ug/ul protein) were de-proteinated with proteinase K (0.4 ug/ul final) at 60°C for 1 hour and re-boiled prior to resolution through 4-12 % gradient SDS-PAGE pre-cast gels (Invitrogen). Each lane was loaded with the de-proteinated material generated from *F. tularensis* lysate containing 10 ug of protein. Carbohydrates were visualized in situ using the Pro-Q Emerald 300 Lipopolysaccharide Gel Stain Kit (Invitrogen) as instructed. Briefly, resolved gels were fixed over-night in 5% acetic acid, 50% methanol. Following two 20 minute washes in 3% acetic acid, the gels were incubated for 30 minutes in oxidizing solution, washed 3 times in 3% acetic acid prior to fluorescent staining of oxidized carbohydrates. After two 20 minute washes in 3% acetic acid, the gels were visualized using a SYBR-green filter and an Alpha Innotech imaging system in movie mode. Following acquisition of the emerald-green signal, gels were de-stained overnight in 3% acetic acid and subsequently stained with SYPRO Ruby fluorescent protein stain (Invitrogen) and visualized using an ethidium bromide filter as above.

### Antibody- and complement-binding assays

To assess binding of Ab or complement to the surface of *F. tularensis,* 5×10^8^ bacteria in 25 ul were mixed with 25 µl of sterile, pre-cleared (10,000 x *g* for 10 min) 0.5 X PBS containing 0.1% glucose and either **i)** 1–5 µl (empirically-determined) of the test Ab/sera (sera were heat-inactivated) for Ab-binding assays or **ii)** 12.5 µl of intact or heat-inactivated normal mouse sera (for complement-binding assays). To assess the impact of protease inhibitors on Ab-binding, a protease inhibitor cocktail (Sigma, p2714 – which includes aprotinin, a plasmin inhibitor) was included in the above Ab mixture. Samples were Incubated at 37°C for 1.5 hrs (for Ab-binding assays) or for various times (10 min – 1 hr) for complement-binding assays. Following incubation, bacteria were pelleted by centrifugation (10,000 x *g* for 10 min) and 45 µl of supernatant was removed. The bacteria were gently resuspended in 1 ml of 0.5 X PBS containing 0.1% glucose and pelleted by centrifugation (10,000 x *g* for 10 min) after which the supernatants were aspirated. Following one additional wash, the cells were resuspended to 25 µl in 50 mM Tris pH 8.0 containing protease inhibitors followed by the addition of 25 µl of sample buffer prior to boiling and SDS-PAGE. 1×10^8^ bacteria per lane were resolved by SDS-PAGE and probed for bound immunoglobulin (IgG or IgM) heavy chain (HC) by western blot. Following development of the Ig HC signals, we re-probed the membranes for total FopA and quantified the data as surface Ab/total FopA and normalized the ratios to the corresponding MHB result. Statistical analysis was performed with the 2-tailed, T-test with Bonferonni corrections when appropriate and significance set at *p*<0.05. As a control, we included a BHI-grown *wbtA* strain [114] since such strains produce neither LPS OAg nor capsular OAg [30]. Normal mouse serum (NMS) and α-IglC Ab (directed against a sub-surface protein) were used as control Abs.

### Macrophage infections for cytokine analysis

BMDM seeded in 24 or 48 well plates were co-incubated at an MOI of 100 with *F. tularensis* that had been grown in vitro (MHB, BHI, MS, BCA) or harvested from previously-infected BMDM. After 24 hrs the media was analyzed by ELISA (eBiosciences) to measure TNF-α and Cytometric Bead Array (BD Pharmingen) to detect IL-1β. Statistical analysis was conducted using GraphPad Prism and one-way ANOVA or the Students T-test with Bonferonni corrections when appropriate and significance set at *p*<0.05.

### Ethics Statement

All animals were handled in strict accordance with good animal practice as defined by the relevant national and/or local animal welfare bodies, and all animal work was approved by the Albany Medical College Animal Care and Use committee (Approval # 901398). Guidelines provided by the NIH were followed in all experimentation.

### Murine infections

We generated two independent sets of matched challenge stocks (MHB- and BHI-grown *F. tularensis* SchuS4). Each matched set was generated from a common pool of single isolated colonies, picked from a single chocolate ager plate and used to inoculate both MHB and BHI broth; each broth culture was expanded side-by-side and harvested at O.D._600_ = 0.2. In three independent experiments totaling 26–28 mice per media type, groups consisting of 6–10 C57BL/6 mice, 8–12 wks of age, were anesthetized by intra-peritoneal injection of 100 µl of xylazine (20 mg/ml) and ketamine (1 mg/ml) and challenged by intranasal instillation of ∼20 CFU of MHB- or BHI-grown *F. tularensis* SchuS4 in 20 µl of PBS. Exact CFUs/inoculum were determined by duplicate plating of the inoculums at the time of challenge. Mice were monitored once-to-thrice daily until all mice succumbed to infection (here less than 8 days). Two of the three experiments used one matched set of challenge stocks; the other experiment used the other set of matched challenge stocks. Analysis of the survival data by the Cox proportional hazard model determined that **i)** there were no significant differences among the three experiments (*p*>0.79) and **ii)** the small variation in inoculm size (21–26 CFU) had no significant impact (*p*>0.68). This analysis did however find that inoculum type (MHB- or BHI-grown *F. tularensis* SchuS4) had a significant (*p* = 0.0046) impact on survival with a hazard ratio for BHI-grown bacteria of 2.38 (95% C.I. of 1.31–4.33). This indicates that at any point in the experiment, a mouse infected with BHI-grown SchuS4 was 2.4 times more likely to die as a mouse infected with MHB-grown SchuS4. The observation that the three experiments were not significantly different also allowed for analysis of the combined survival data from all 54 mice by the Kaplan-Meier log-rank test.

## Supporting Information

Figure S1 **The cellular distribution of the** ***F. tularensis*** **KatG, GroEL, SodB, and Bfr proteins is markedly distinct from that of a secreted protein (** ***E. coli*** **HlyA).** *F. tularensis* LVS and *E. coli* WAM 1824 cultures grown in MHB (M) or BHI (B) to mid-log were quantified and harvested by centrifugation. Sterile-filtered, cell-free supernatants from 5×10^9^ bacteria were precipitated with 10 volumes of acetone, washed twice with 70 % ethanol and resuspended in 1% SDS. Cells and supernatants from the indicated number of bacteria were resolved by SDS-PAGE and either stained with coomassie blue (top panels) or analyzed by western blot (lower panels) for the indicated proteins. Note that the immunoblot signal intensity for MHB-grown *F. tularensis* is similar in 10^7^ cells and 10^9^ supernatant equivalents indicating that ∼1% of each protein is found in the MHB supernatant. Expression of HlyA is environmentally-regulated and, in uropathogenic *E. coli*, is increased during mammalian infection.
(TIF)

Figure S2 **Fractionation of** ***F. tularensis*** **reveals the presence of an inducible HMW carbohydrate in host-adapted bacteria.** Whole cells (WC) of *F. tularensis* LVS grown in MHB (M) or BHI (B) were Tx114 phase-partitioned into aqueous (A), detergent (D), and insoluble fractions (TxI). The TxI material was treated with 0.2% sarkosyl (S) resulting in soluble (SS) and insoluble (SI) fractions. Following proteinase-K treatment and SDS-PAGE resolution, the samples were stained for carbohydrates. Different exposures (top panel-short exposure, bottom panel- longer exposure) of the same gel are shown here and in Figure 2B. Western blots with mAb FB11 (specific for LPS OAg, data not shown) confirmed the identification of the band labeled “LPS core + 1 OAg unit”.
(TIF)

Figure S3 ***F. tularensis*** **grown in BHI produces a both an OAg and non-OAg HMW carbohydrate and a ∼200 kDa putative glycoprotein that partition into the Tx-114 aqueous phase.** Tx114 aqueous phases from MHB (M)- and BHI (B)-grown *F. tularensis* WT and *wbtA* were treated with proteinase K (PK) and resolved by SDS-PAGE. The resolved samples were sequentially stained to visualize carbohydrates (left panel) followed by visualization of protein (right panel). Note that the bottoms of the loading wells are visible.
(TIF)

Figure S4 **Host-adaptation of** ***F. tularensis*** **reduces complement activation**. MHB- or BHI-grown bacteria were incubated for 10 min or 1 hr with 25% normal mouse serum (NMS), heat-inactivated (hi) NMS, or in the absence of NMS. Washed bacteria were probed by western blot with a polyclonal Ab directed against mouse complement protein C3; the α-C3 Ab was found to be selective for the α-chain of C3. Results are representative of three independent experiments.
(TIF)
