# Supplementary figures and images for: Host-Adaptation of Francisella tularensis Alters the Bacterium's Surface-Carbohydrates to Hinder Effectors of Innate and Adaptive Immunity
Source: PLoS One. 2011 Jul 22;6(7):e22335. doi: 10.1371/journal.pone.0022335 (PMC3142145; doi:10.1371/journal.pone.0022335)

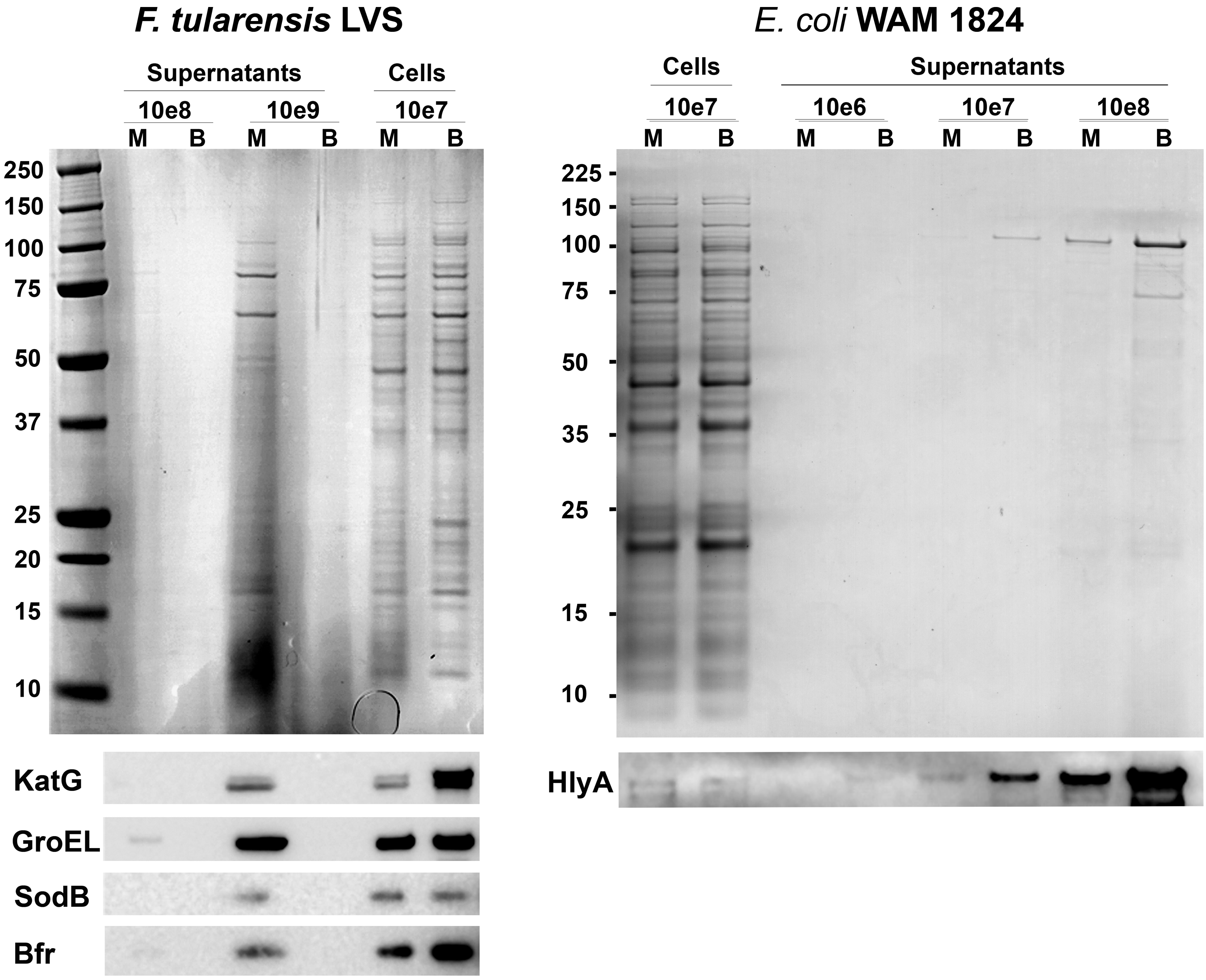

Supplement: Figure S1 — The cellular distribution of the F. tularensis KatG, GroEL, SodB, and Bfr proteins is markedly distinct from that of a secreted protein ( E. coli HlyA). F. tularensis LVS and E. coli WAM 1824 cultures grown in MHB (M) or BHI (B) to mid-log were quantified and harvested by centrifugation. Sterile-filtered, cell-free supernatants from 5×10^9^ bacteria were precipitated with 10 volumes of acetone, washed twice with 70 % ethanol and resuspended in 1% SDS. Cells and supernatants from the indicated number of bacteria were resolved by SDS-PAGE and either stained with coomassie blue (top panels) or analyzed by western blot (lower panels) for the indicated proteins. Note that the immunoblot signal intensity for MHB-grown F. tularensis is similar in 107 cells and 109 supernatant equivalents indicating that ∼1% of each protein is found in the MHB supernatant. Expression of HlyA is environmentally-regulated and, in uropathogenic E. coli, is increased during mammalian infection. (TIF) [file pone.0022335.s001.tif]

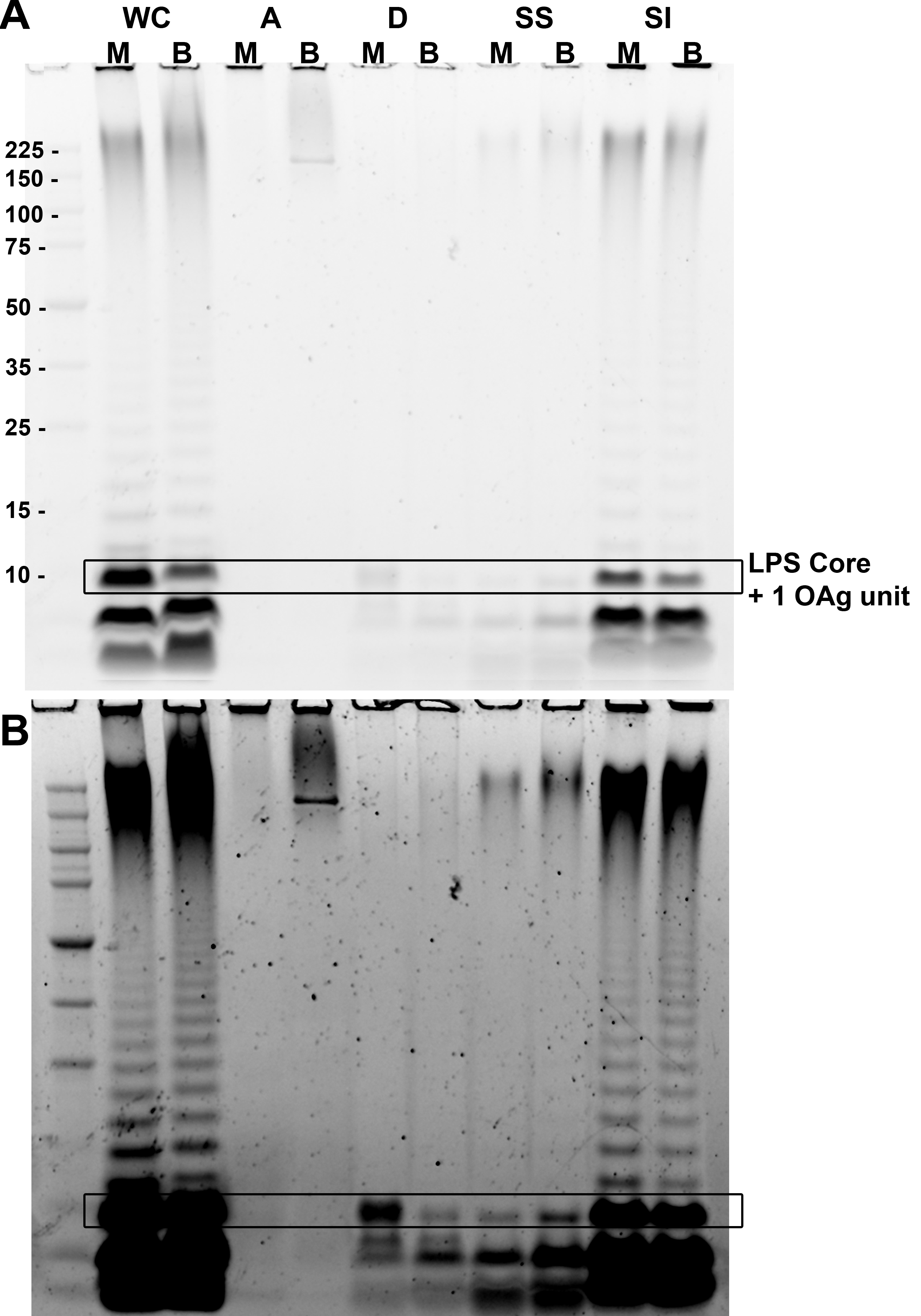

Supplement: Figure S2 — Fractionation of F. tularensis reveals the presence of an inducible HMW carbohydrate in host-adapted bacteria. Whole cells (WC) of F. tularensis LVS grown in MHB (M) or BHI (B) were Tx114 phase-partitioned into aqueous (A), detergent (D), and insoluble fractions (TxI). The TxI material was treated with 0.2% sarkosyl (S) resulting in soluble (SS) and insoluble (SI) fractions. Following proteinase-K treatment and SDS-PAGE resolution, the samples were stained for carbohydrates. Different exposures (top panel-short exposure, bottom panel- longer exposure) of the same gel are shown here and in Figure 2B. Western blots with mAb FB11 (specific for LPS OAg, data not shown) confirmed the identification of the band labeled “LPS core + 1 OAg unit”. (TIF) [file pone.0022335.s002.tif]

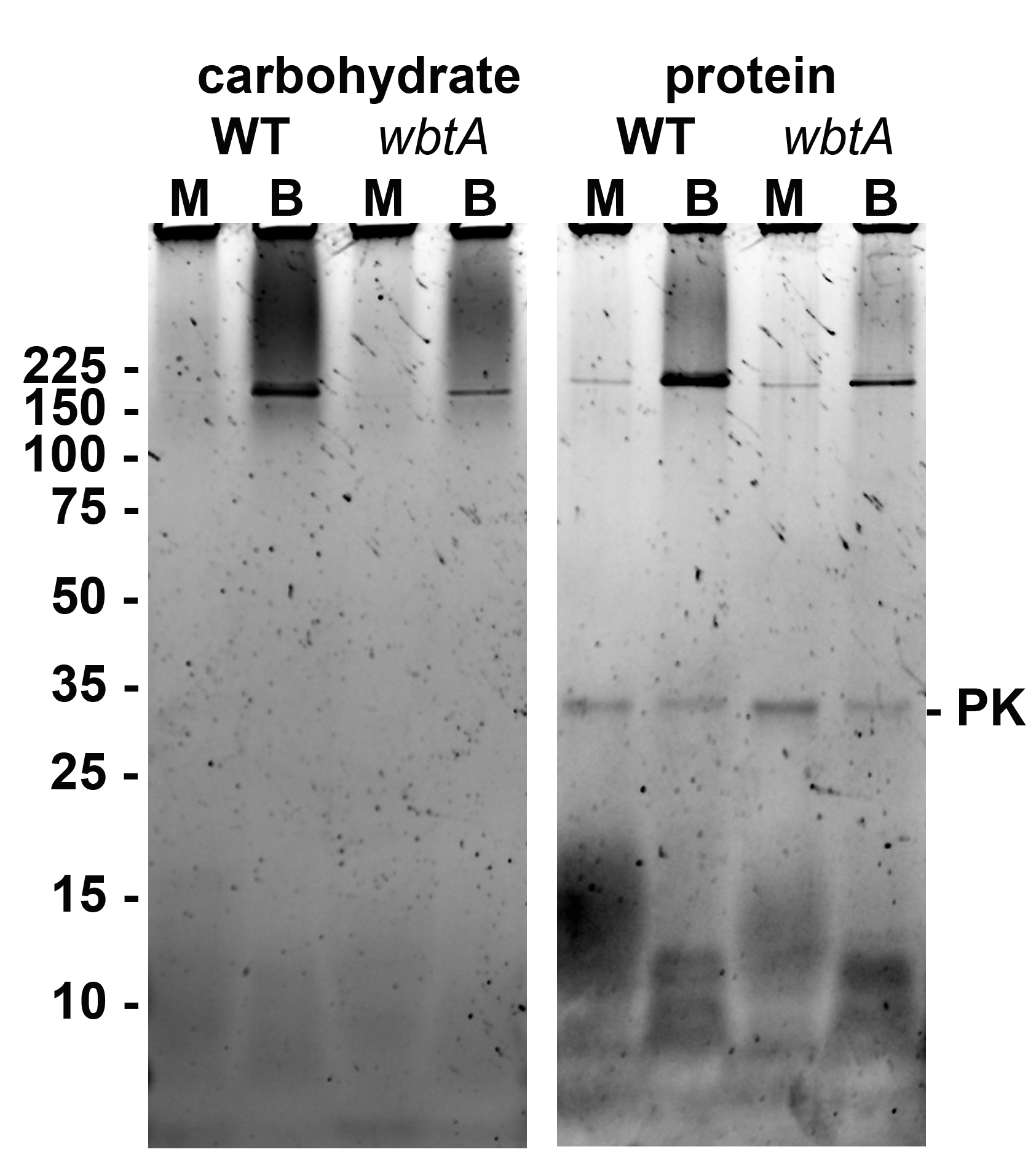

Supplement: Figure S3 — F. tularensis grown in BHI produces a both an OAg and non-OAg HMW carbohydrate and a ∼200 kDa putative glycoprotein that partition into the Tx-114 aqueous phase. Tx114 aqueous phases from MHB (M)- and BHI (B)-grown F. tularensis WT and wbtA were treated with proteinase K (PK) and resolved by SDS-PAGE. The resolved samples were sequentially stained to visualize carbohydrates (left panel) followed by visualization of protein (right panel). Note that the bottoms of the loading wells are visible. (TIF) [file pone.0022335.s003.tif]

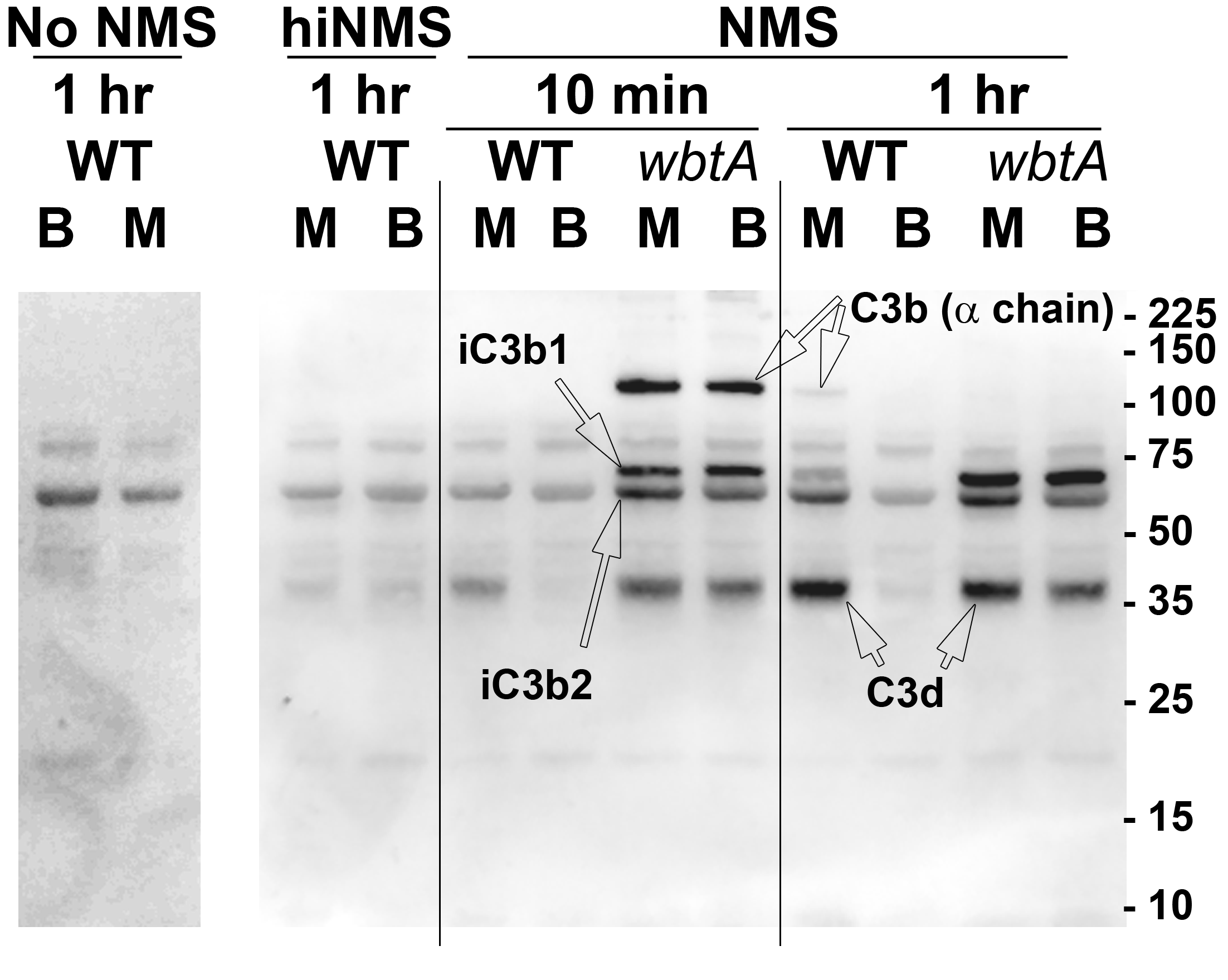

Supplement: Figure S4 — Host-adaptation of F. tularensis reduces complement activation. MHB- or BHI-grown bacteria were incubated for 10 min or 1 hr with 25% normal mouse serum (NMS), heat-inactivated (hi) NMS, or in the absence of NMS. Washed bacteria were probed by western blot with a polyclonal Ab directed against mouse complement protein C3; the α-C3 Ab was found to be selective for the α-chain of C3. Results are representative of three independent experiments. (TIF) [file pone.0022335.s004.tif]
